# The use of herbal medicines by people with cancer: a cross-sectional survey

**DOI:** 10.1038/bjc.2011.47

**Published:** 2011-03-01

**Authors:** S Damery, C Gratus, R Grieve, S Warmington, J Jones, P Routledge, S Greenfield, G Dowswell, J Sherriff, S Wilson

**Affiliations:** 1 Primary Care Clinical Sciences, School of Health and Population Sciences, University of Birmingham, Edgbaston, Birmingham B15 2TT, UK; 2 Arden Cancer Research Centre, University Hospitals Coventry and Warwickshire NHS Trust, Clifford Bridge Road, Coventry CV2 2DX, UK; 3 Section of Pharmacology, Therapeutics and Toxicology, Cardiff University, Heath Park, Cardiff CF14 4XN, UK

**Keywords:** herbal medicines, survivorship, prevalence

## Abstract

**Background:**

A large proportion of cancer patients are estimated to use herbal medicines, but data to substantiate this are lacking. This study aimed to investigate the prevalence of herbal medicine use among cancer patients in the West Midlands, and determine the characteristics predicting herbal medicine use.

**Methods:**

A cross-sectional survey of oncology patients (*n*=1498) being followed up at a hospital in Coventry was undertaken. Recipients were asked about herbal medicine use since their cancer diagnosis, and the association between sociodemographic and cancer-related characteristics and herbal medicine use was evaluated.

**Results:**

A total of 1134 responses were received (75.7%). The prevalence of herbal medicine use was 19.7% (95% CI: 17.4–22.1; *n*=223). Users were more likely to be affluent, female, and aged under 50 years. Usage increased with time since cancer diagnosis (*X*^2^ for trend=4.63; *P*=0.031). A validation data set, derived from a survey of oncology patients in Birmingham (*n*=541) with differing socioeconomic characteristics showed no significant difference in estimated prevalence (16.6% 95% CI: 11.9–22.2).

**Conclusion:**

A substantial number of people with cancer are likely to be taking herbal medicines. Understanding the self-medication behaviours of these individuals is essential if health-care professionals are to support treatment adherence and avoid unwanted pharmacological interactions.

Between 9 and 81% of cancer patients are said to use at least one type of complementary or alternative therapy after their cancer diagnosis (Ernst, 2000; Catt *et al*, 2006). In particular, self-medication with herbal medicines and other ‘natural’ substances is widespread and increasing in the United Kingdom (Werneke *et al*, 2004a; Astin *et al*, 2006). It has been estimated that a large proportion of patients with cancer use herbal medicines. These may be taken to prevent or relieve some of the symptoms of the cancer itself (Ali and Hussain-Gambles, 2005); to help alleviate some of the side effects from cancer treatments, such as radiotherapy or chemotherapy (Vickers *et al*, 2006); to treat an associated condition, such as anxiety or depression, or to provide a sense of control or a feeling of active involvement in cancer treatment (Verhoef *et al*, 1999). However, estimates of the prevalence of herbal medicine use are inconsistent (varying from 3 to 25% of cancer patients), and the quality and scope of existing studies are limited (Ernst and Cassileth, 1998; Gratus *et al*, 2009a). Research typically focuses on restricted patient cohorts and specific tumour sites; includes small numbers of participants, and uses heterogeneous methodologies and definitions of herbal medicines, which make it difficult to assess the precise extent of herbal medicine use by cancer patients (Crocetti *et al*, 1998). Herbal medicines are also frequently subsumed under the broader heading of complementary and alternative medicines, and their use assessed alongside other therapies, such as aromatherapy, reflexology, meditation, acupuncture, and homeopathy (Downer *et al*, 1994; Corner *et al*, 2006).

Among cancer patients, users of herbal medicines tend to be female, younger, and have higher socioeconomic status than non-users (Harris *et al*, 2003). Disease-related factors, such as the type of cancer, stage of disease, and disease duration have also been found to be significant predictors of herbal medicine use (Miller *et al*, 1998), with women with breast cancer most likely to use herbal medicines in comparison with the general population, and compared with those with other cancer types (Morris *et al*, 2000). However, studies of breast cancer patients and herbal medicine use constitute the majority of research literature, and may not be representative of other cancer diagnostic groups (Molassiotis *et al*, 2005).

Alongside increased interest in the use of herbal medicines by people with cancer, there has been a rise in concern about the safety of these treatments (Balneaves *et al*, 1999). Herbal medicines are often seen as more natural, and therefore safer than conventional treatments, and it is generally believed that they carry little potential for harm (Corner *et al*, 2006; Vickers *et al*, 2006). However, in some cases, herbal medicines can present significant risks (Gratus *et al*, 2009a, 2009b). They may affect adherence with prescribed treatments, cause harmful interactions with conventional medications, reduce treatment efficacy, or lead to adverse events (Frye *et al*, 2004; Catt *et al*, 2006; Medicines and Healthcare Products Regulatory Agency, 2007). A recent systematic review of herbal medicine use by cancer patients identified 21 case reports of toxic effects and adverse events in users of herbal medicines (Olaku and White, 2010). As a result of the possibility of interactions between herbal medicines and conventional treatments, people with cancer are encouraged to advise health-care professionals if they are taking any type of medication, including herbal medicines and other supplements (Cancer Backup, 2010), although studies suggest that few patients do so (Corner *et al*, 2006).

Given the potential risks, and the lack of data to substantiate the use of herbal medicines by cancer patients in the United Kingdom, there is a need to better understand the prevalence of herbal medicine use among cancer patients, as well as improving knowledge of which herbal medicines patients use. This is an important first step in the development of accessible, authoritative, and independent information resources about herbal medicines and cancer, which are currently lacking in the United Kingdom (Gratus *et al*, 2009b). The aim of this study was to investigate the prevalence of herbal medicine use among people with cancer in the West Midlands, and to determine the sociodemographic and cancer-related characteristics that may predict herbal medicine use within this group.

## Materials and methods

### Study design and setting

This study used a cross-sectional survey, distributed by post, to oncology patients being followed up at University Hospitals Coventry and Warwickshire NHS Trust (UHC&W).

### Participants and recruitment

Subsequent to securing permission from consultants responsible for the care of patients, the hospital information system was interrogated to identify a sample of eligible patients aged 18 and over, at least 6 months and no >5 years after a diagnosis of invasive cancer (between 1 April 2004 and 30 June 2009), who had been treated with curative intent. A total of 1507 potential participants were identified. The responsible consultant examined the list of potential participants to verify that the patient had been treated for invasive cancer and had been diagnosed within the specified time frame. Patients known to be terminally ill, or whom the consultant believed may be distressed by receipt of the survey for any other reason, were excluded from the sample to minimise the possibility of distress, as were any individuals whose records did not indicate a consultant; duplicate records; those for whom a valid postal address could not be identified, and those who had died in the period between sample identification and survey mailing.

After exclusions had been made, 1498 eligible patients were sent a letter of invitation, a patient information sheet and a survey, to be returned to the research team at University of Birmingham via a FREEPOST envelope, also enclosed. This sample size assumed a prevalence of herbal medicine use among cancer patients of between 7 (Corner *et al*, 2006) and 13% (Harrison *et al*, 2004), and that a 60% response rate would yield 900 responses. This sample would be of sufficient size to determine the overall prevalence of herbal medicine use among respondents with a precision of 2% (95% confidence interval). Survey recipients who had not responded to the initial mailing after 2 weeks received one reminder.

### Survey

Survey content was informed by the findings of a systematic review of the literature relating to herbal medicine use by cancer patients in the United Kingdom (Gratus *et al*, 2009a). The survey included closed questions on sociodemographic characteristics (age, gender, and ethnicity), cancer-related characteristics (year of diagnosis), and a number of categorical (yes/no response) questions relating to the use of herbal medicines, vitamin/mineral supplements and homeopathic remedies; the sources through which herbal medicines were obtained by users (e.g., bought on the high street, from a herbal medicine practitioner, purchased via the Internet), and the use of specific herbal medicines since diagnosis, detailed in a pre-coded ‘tick-list’, derived from a review of the most commonly cited herbal medicines from existing research literature. Patients were able to select multiple herbal medicines if they had used more than one. They were also able to list any additional herbal medicines that they had used since diagnosis, which were not included in the pre-coded list, using a free-text response box.

### Data analysis

Analysis focused on the sociodemographic and cancer-related characteristics of respondents, and their association with herbal medicine use. In addition to the data obtained from respondents via their survey responses, anonymised hospital records for each respondent were used to derive information related to cancer type, classified according to International Classification of Diseases (ICD-10) categories for invasive cancer (World Health Organization, 1994), and postcode data were used to derive a deprivation score, which was converted into a deprivation quartile using the 2007 Indices of Multiple Deprivation: quartile 1=most affluent; quartile 4=most deprived (Department of Communities and Local Government, 2007).

An individual was defined as a herbal medicine user if they answered ‘yes’ to the question: ‘have you used herbal remedies since your cancer was diagnosed?’ and/or gave a positive response to any question asking about herbal medicine purchasing, and/or indicated the use of a specific herbal medicine from the pre-coded list or via the free-text response box. Free-text responses were coded and analysed in the same way as herbal medicines indicated in the tick-list. Chi-squared tests and binary logistic regression were used to compute bivariate and multivariate odds ratios (ORs) to evaluate the association between sociodemographic and cancer-related characteristics and herbal medicine use, and to assess the factors predictive of herbal medicine use by patients with cancer. In order to test for responder bias, age, gender, ethnicity, deprivation, and time since diagnosis distributions for respondents and non-respondents were compared using *χ*^2^ tests. Prevalence rates of herbal medicine use were age and gender standardised to the cancer patient population in England using the 2006 cancer registration statistics (Office for National Statistics, 2006). All data were analysed using SPSS (version 15.0, SPSS Inc., Chicago, IL, USA).

### Validation data set

As the survey population was identified from the records of one hospital only, the survey was repeated on a smaller scale at another hospital in Birmingham (541 patients mailed), in order to validate the rates of herbal medicine use identified from survey respondents treated at UHC&W and assess the wider generalisability of the findings. Patients were identified at the participating hospital in Birmingham in the same way as for the larger survey, according to the same eligibility criteria, and received the same survey and study literature; however, non-responders did not receive a reminder.

## Results

**Figure 1:**
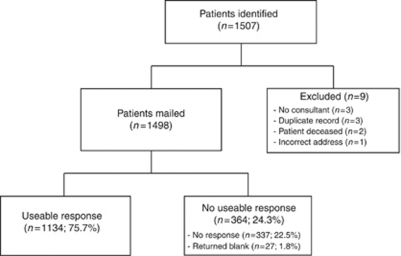
Consort diagram detailing surveys mailed and returned.

Of 1498 surveys distributed, 27 (1.8%) were returned blank, indicating that the recipient did not wish to receive a reminder. A further 337 survey recipients (22.5%) did not respond to either the initial or reminder mailings, giving a total of 1134 useable responses (response rate 75.7%), Figure 1.

Some statistically significant differences were found between the sociodemographic and cancer-related characteristics of responders and non-responders. Those in the youngest age group (<50 years old) were significantly less likely to respond than those in older age groups (*X*^2^=21.28; *P*=<0.0001), and those of non-White ethnicity were less likely to respond than those in the White ethnic group (*X*^2^=105.48; *P*=<0.0001). Finally, those in the most deprived deprivation quartiles were significantly less likely to respond to the survey than those in more affluent quartiles (*X*^2^=45.61; *P*=<0.0001). There were no significant differences between responders and non-responders on the basis of gender (*X*^2^=1.87; *P*=0.172) or the number of years since a patient had received their diagnosis of cancer (*X*^2^=1.12; *P*=0.571).

### Characteristics of respondents

The sociodemographic and cancer-related characteristics of respondents are shown in Table 1. The majority of respondents were female (*n*=821; 72.4%), and in the White ethnic group (*n*=1078; 95.1%). Patients aged between 60 and 69 years old constituted the largest group (*n*=382; 33.7%), with those aged under 50 forming the smallest respondent group (*n*=188; 16.6%). Patients in the two most affluent deprivation quartiles (quartiles 1 and 2) constituted 60.3% of respondents (*n*=681), compared with 15.6% of respondents (*n*=176) in the most deprived quartile. With regard to cancer-related characteristics, the greatest proportion of respondents had received their diagnosis of cancer between 2 and 4 years before the survey mailing (*n*=460; 40.6%). Patients with breast cancer constituted the largest group according to cancer type, accounting for over half of all respondents (*n*=585; 51.6%), followed by 14.1% with cancer related to the digestive organs (*n*=160), and male genital cancers (*n*=151; 13.3%). The least represented cancer types were amalgamated and categorised as ‘other’ cancers (*n*=23; 2.0%). This included primary bone cancer (*n*=1), soft tissue sarcomas (*n*=4), and cancers of the skin (*n*=3), urinary tract (*n*=12), eye, brain, and central nervous system (*n*=1), and cancers of unknown origin (*n*=2).

### Prevalence of herbal medicine use

Across all respondents, the crude prevalence of herbal medicine use was 19.7% (95% CI: 17.4–22.1; *n*=223). The age and gender standardised prevalence rate, calculated using the 2006 cancer registrations statistics for England was 16.8%. A total of 282 patients had used vitamin supplements (24.9%), 258 had used mineral supplements (22.8%), 59 had used some form of homeopathic remedies (5.2%), and 176 had used other complementary and alternative therapies, such as aromatherapy or massage (15.5%).

Of the herbal medicine users (*n*=223), 55.2% (*n*=123) had used vitamin supplements in addition to herbal medicines, 49.3% (*n*=110) had used mineral supplements as well as herbal medicines, 21.5% (*n*=48) had also used homeopathic remedies, and 41.2% had used other complementary and alternative therapies. Six individuals (2.7%) reported using all of these.

Univariate analyses indicated that a greater proportion of female respondents reported using herbal medicines than males (*n*=183, 22.3% *vs n*=40, 12.8%), Table 1. Respondents in the White and non-White ethnic groups did not differ in their use of herbal medicines (*n*=212, 19.7% *vs n*=11, 19.6%). Respondents in the 50–59 year age group had the highest prevalence of herbal medicine use (*n*=61; 25.0%), as did those in the two most affluent deprivation quartiles (*n*=144; 21.1%). Patients who were >4 years since diagnosis had the highest rate of use (*n*=64; 24.1%), and patients with female genital cancers and breast cancer were most likely to be herbal medicine users (*n*=28; 24.6% *vs n*=133; 22.7%).

### Predictors of herbal medicine use

Binary logistic regression was used to calculate bivariate OR to evaluate the association between sociodemographic or cancer-related respondent characteristics and herbal medicine use (Table 1). Users of herbal medicines were significantly more likely to be in the two most affluent deprivation quartiles in comparison with the most deprived quartile (quartiles 1 and 2, OR: 1.7; 95% CI: 1.1–2.7). A *χ*^2^ test for trend confirmed a decreasing likelihood of herbal medicine use with increasing deprivation (*X*^2^=4.13; *P*=0.042). Females were nearly twice as likely to be herbal medicine users as males (OR: 1.9, 95% CI: 1.4–2.8; *P*=<0.0001). Herbal medicine users were also more likely to be younger, with patients aged under 50 years old significantly more likely to use them than those in the 70+ age group (OR: 1.6; 95% CI: 1.0–2.5), and those aged between 50 and 59 having the highest likelihood of using herbal medicines (OR: 1.8; 95% CI: 1.2–2.7; *P*=0.006). There was no association between ethnic group and herbal medicine use.

With regard to cancer-related characteristics, people with breast cancer were most likely to be herbal medicine users, with all other cancer types except female genital cancers having a lower usage. Those with oral and respiratory cancers (including lip, oral cavity, head and neck and lung cancers) were significantly less likely than people with breast cancer to use herbal medicines (OR: 0.3; 95% CI: 0.1–0.9), as were patients with thyroid and lymphoid cancers (OR: 0.3; 95% CI: 0.1–0.8; *P*=0.016). Usage increased with time since diagnosis, with patients over 4 years since diagnosis the most likely to be herbal medicine users (*X*^2^ for trend=4.63; *P*=0.031).

Logistic regression was also used to calculate multivariate OR, with each variable in the model controlled for all other variables. All significant predictive factors for herbal medicine use observed in the bivariate analysis (affluence, younger age, longer time since diagnosis, and female gender) remained significant in the multivariate model, with the exception of cancer type.

### Use of specific herbal medicines

All herbs detailed in the pre-coded survey list were used by at least one individual, with the exception of Ivy (*Hedera helix*), Table 2. Evening Primrose (*Oenothera biennis*) was the most frequently used herb (*n*=61; 27.4%), followed by Echinacea (*Echinacea purpurea*) (*n*=48; 21.5%) and Garlic (*Allium sativum)* (*n*=43; 19.3%). Breast cancer patients were the most likely to use each of the specified herbs, constituting 100% of users of Agnus castus (*Vitex agnus castus*), Dong quai (*Angelica sinensis*), Red vine leaf (*Vitis vinifera*), Wild Yam (*Dioscorea villosa*), and Willow (*Salix alba*). Of the other herbs, only Saw palmetto (*Serenoa serrulata*) was used exclusively by patients within one cancer type – in this case, all users (*n*=3) were in the male genital cancer group.

Patients were also given the opportunity to cite the use of other herbal medicines not detailed in the pre-coded list. Nineteen additional herbal medicines were reported in this way, of which only six were used by more than one individual: herbal teas (*n*=14); Aloe vera (*Aloe barbadensis*), (*n*=8); Arnica (*Arnica montana*), (*n*=6); Starflower (*Borago officinalis*), (*n*=4); Sage (*Salvia officinalis*), (*n*=3), and Turmeric (*Curcuma longa*), (*n*=3).

### Where herbal medicines were obtained

The majority of herbal medicine users reported obtaining their herbal medicines from high street stores and supermarkets (*n*=151; 67.7%). Recommendation by a health-care professional was a frequent source of information (*n*=52; 23.3%), as was purchase of herbal medicines from the Internet or through mail order (*n*=51; 22.9%). Users were less likely to obtain herbal medicines following a consultation with a herbal practitioner; taken together, 18 unique individuals had consulted a practitioner of Western herbal medicine, Chinese/Ayurvedic herbal medicine, or a practitioner from another herbal medicine tradition (8.1%), Table 3.

### Validation data set

To validate the prevalence estimates for herbal medicine use obtained from patients of UHC&W, a further small-scale survey was undertaken involving people with cancer being followed up at a hospital in Birmingham. In all, 217 individuals responded to this survey from 541 mailed (response rate 40.1%). Respondents in Birmingham differed from respondents to the larger survey with regard to a number of characteristics. University Hospitals Coventry and Warwickshire NHS Trust respondents were significantly more likely to be female than those from Birmingham (OR: 1.5; 95% CI: 1.3–1.7; *P*=<0.0001). They were also twice as likely to be in the more affluent deprivation quartiles (OR: 2.1; 95% CI: 1.7–2.6; *P*=<0.0001), and were a younger population (*X*^2^=17.56; *P*=0.001). Respondents from UHC&W were less likely to be in the non-White ethnic group than those from Birmingham (OR: 0.5; 95% CI: 0.3–8.2; *P*=0.006).

The prevalence of herbal medicine use in the group from Birmingham was 16.6% (95% CI: 11.9–22.2; *n*=36). Despite the differences in the nature of the respondent populations to both surveys, there was no statistically significant difference in the prevalence of herbal medicine use between the two populations surveyed (*X*^2^=1.11; *P*=0.292).

## Discussion

Almost one-fifth of our survey respondents reported using herbal medicines in the time since their diagnosis of cancer. Age and gender standardisation of this prevalence rate to the England cancer patient population gave a standardised prevalence estimate of 16.8%. With around two million people in the United Kingdom currently living with cancer, this could equate to approximately 336 000 individuals with cancer who are regular users of herbal medicines in the United Kingdom.

By focusing on herbal medicines specifically, rather than subsuming them under the broader term of complementary and alternative therapies, this study adds to the very limited existing literature regarding herbal medicine use by cancer patients in the United Kingdom (Gratus *et al*, 2009a). Many studies of this nature involve small numbers of participants and so cannot provide precise estimates of herbal medicine use (Rees *et al*, 2000). The response rate for our survey was high, and the sample size was large (75.7% *n*=1134). The findings have face validity and fit well with other research, which has found that women, those in younger age groups, and those who are more affluent are the most likely subgroups of cancer patients to use herbal medicines (Ernst and Cassileth, 1998; Harris *et al*, 2003; Catt *et al*, 2006). A large proportion of users were likely to use other complementary and alternative therapies in addition to herbal medicines, such as vitamins, mineral supplements, homeopathic remedies, or other therapies, such as aromatherapy or massage.

Although some herbal medicines are used only by those with particular cancer types, such as Saw palmetto (*Serenoa serrulata*) used exclusively by patients with male genital cancers (Olaku and White, 2010), herbal medicines were typically used by our respondents across a range of cancer types, suggesting that the majority of herbs do not seem to be targeted towards specific cancer sites, but that they may be used for a range of reasons, such as the reduction of symptoms associated with the cancer or its treatment, or to address associated conditions or co-morbidities.

In common with other studies, we found that women with breast cancer are particularly likely to use herbal medicines, as are women with genital cancers, although in the multivariate analyses, cancer site was not a significant predictor of herbal medicine use. This may reflect the fact that for some cancer types, there may be limited herbal medicines available, particularly if these are being sought to alleviate specific conditions or side effects, either when patients are undergoing treatment or are in the post-treatment phase. We also found that the likelihood that people with cancer are using herbal medicines increases with time since diagnosis. Lower rates of use in the first 2 years after diagnosis are plausible because patients are likely to be undergoing conventional treatments, such as radiotherapy or chemotherapy at this time. Higher usage in the years following treatment may indicate that patients were more likely to consider using herbal medicines to address the long-term consequences of cancer treatment. This suggests that there may be less of a need to be concerned about possible harmful interactions with conventional treatments than is often asserted (McCune *et al*, 2004). However, there are documented interactions between some herbal medicines and other conventional medicines, such as warfarin (Ali and Hussain-Gambles, 2005) or tamoxifen (Werneke *et al*, 2004b), and those taking prescribed medication for other co-morbidities may experience harmful interactions when using herbal medicines.

It is important that information resources regarding herbal medicines and their safety and efficacy are available to both ongoing patients and to cancer survivors. Furthermore, if patients require further treatment, following recurrence of disease, for example, it is possible that herbal medicine use has become established by this time. Routine clinical questioning should encompass herbal medicine use before all treatment episodes.

### Limitations

This study had several limitations. First, prevalence surveys that rely on patient-reported use of herbal medicines may be subject to recall bias. This may be deliberate, in that cancer patients may not disclose herbal medicine use, particularly if they have not told the health-care professionals treating them about any herbal medicines or supplements that they may be using (Evans *et al*, 2007; Saxe *et al*, 2008). Recall bias may also be inadvertent, where patients may not remember whether or not they have taken herbal medicines since their cancer diagnosis, and consequently either under- or over-report their herbal medicine use. With respect to the issue of non-disclosure, patients were informed that all survey responses would be kept confidential and that members of their medical team would not see any of the information that they supplied in response to the survey.

Second, responder bias may mean that we have either under- or over-estimated the prevalence of herbal medicine use by cancer patients in our study population. Responders to the survey were more likely to be affluent, older, and in the White ethnic group, although the proportion of survey respondents in the White and non-White ethnic groups was broadly representative of the ethnic mix of oncology patients in England (National Cancer Intelligence Network, 2009). However, by age and gender standardising our prevalence estimate to the England cancer patient population, we have derived a reliable estimate of herbal medicine use by cancer survivors.

To minimise the risk of distress, patients known to be terminally ill or whom the responsible consultant believed could have been distressed by receipt of the survey were excluded from the mailing. It is possible that this group may have been more likely to use herbal medicines to help them cope with the stress they were experiencing, and thus we have under-estimated the overall prevalence of herbal medicine use by cancer patients. However, we have no evidence to support or refute this possibility.

Finally, our survey was conducted with individuals being followed up at a single hospital, which may affect the wider generalisability of the findings. However, alongside the benefits of age and gender standardisation, the inclusion of comparison data derived from repeating the survey on a smaller scale at a hospital in Birmingham, involving a patient population with very different sociodemographic characteristics adds validity to our findings. This is particularly so given that there was no statistically significant difference found between the prevalence rate from the larger survey and the rate observed within the comparison population (19.7% 95% CI: 17.4–22.1 *vs* 16.6% 95% CI: 11.9–22.2; *P*=0.292) despite the comparison population including a greater proportion of older patients, more males, more patients from non-White ethnic groups, and a higher number of socioeconomically deprived patients than the population in UHC&W.

## Conclusions

It is likely that a substantial number of people with cancer are taking herbal medicines at any one time. With such a high number of potential users and the potential for adverse effects, including adverse drug interactions, a robust evidence base for understanding all aspects of herbal medicine use by those with cancer is required. An understanding of the self-medication behaviours of these individuals is essential if health-care professionals are to support treatment adherence and avoid unwanted pharmacological interactions and compromised treatment efficacy. Health professionals need to be aware of which herbal medicines are being taken by their patients. The provision of relevant educational resources for both patients and health professionals is required.

## Figures and Tables

**Table 1:** Characteristics of respondents, herbal medicine use by characteristic and predictors of herbal medicine use

**Characteristic**	**Respondents (%)**	**Herbal medicine users (%)**	**Bivariate OR (95% CI); significance^c^**	**Multivariate OR (95% CI); significance**
*Deprivation* a				
Affluent	681 (60.3)	144 (21.1)	**1.7** (**1.1–2.7); 0.027**	**1.7** (**1.0–2.7); 0.038**
More deprived	273 (24.2)	55 (20.1)	1.6 (0.9–2.6); 0.079	1.5 (0.9–2.6); 0.139
Most deprived	176 (15.6)	24 (13.6)	Reference	Reference
				
*Gender*				
Female	821 (72.4)	183 (22.3)	**1.9** (**1.4–2.8); <0.0001**	**2.2** (**1.1–4.5); 0.031**
Male	313 (27.6)	40 (12.8)	Reference	Reference
				
*Age group*				
<50	188 (16.6)	43 (22.9)	**1.6** (**1.0–2.5); 0.042**	**1.6** (**1.0–2.6); 0.042**
50–59	244 (21.5)	61 (25.0)	**1.8** (**1.2–2.7); 0.006**	**1.8** (**1.2–2.8); 0.008**
60–69	382 (33.7)	69 (18.1)	1.2 (0.8–1.8); 0.39	1.2 (0.8–1.8); 0.391
70+	320 (28.2)	50 (15.6)	Reference	Reference
				
*Ethnic group*				
White	1078 (95.1)	212 (19.7)	1.0 (0.5–1.9); 0.997	1.1 (0.5–2.2); 0.811
Non-White	56 (4.9)	11 (19.6)	Reference	Reference
				
*Cancer type* b				
Oral and respiratory	42 (3.7)	3 (7.1)	**0.3** (**0.1–0.9); 0.027**	0.5 (0.1–1.6); 0.228
Digestive organs	160 (14.1)	26 (16.3)	0.7 (0.4–1.1); 0.078	1.1 (0.6–1.9); 0.743
Other	23 (2.0)	4 (17.4)	0.7 (0.2–2.1); 0.55	1.0 (0.3–3.7); 0.956
Thyroid and lymphoid	59 (5.2)	5 (8.5)	**0.3** (**0.1–0.8); 0.016**	0.4 (0.2–1.1); 0.055
Female genital	114 (10.1)	28 (24.6)	1.1 (0.7–1.8); 0.672	1.2 (0.8–1.9); 0.449
Male genital	151 (13.3)	24 (15.9)	0.6 (0.4–1.0); 0.069	1.6 (0.7–3.7); 0.324
Breast	585 (51.6)	133 (22.7)	Reference	Reference
				
*Years since diagnosis*				
<2	408 (36.0)	70 (17.2)	**0.7** (**0.5–0.9); 0.029**	**0.6** (**0.4–0.9); 0.017**
2–4	460 (40.6)	89 (19.3)	0.7 (0.5–1.1); 0.134	0.7 (0.5–1.0); 0.057
4+	266 (23.5)	64 (24.1)	Reference	Reference
				
All respondents	1134 (100.0)	223 (19.7)		
Age and gender standardised prevalence		16.8%		

Abbreviation: OR=odds ratio.

a Deprivation quartile could not be obtained for four respondents – OR calculated for 1130 respondents only.

b Cancer types include the following: oral and respiratory – lip, oral cavity, head and neck, pharynx, lung and intrathoracic organs; digestive organs – oesophagus, stomach, colorectal, pancreas; thyroid and lymphoid – thyroid and other glands, lymphomas; female genital – uterus, ovaries, vulva; male genital – testicular, prostate; ‘other’ – primary bone cancer, soft tissue sarcomas, skin, urinary tract, eye, brain and central nervous system, unknown primary cancers.

c Bold entries indicate statistically significant associations.

**Table 2:** Frequencies of use since diagnosis for specific herbal medicines, and use by cancer type

		**Number of users by cancer type (%)^a^**						
**Herbal medicine (Latin name)**	**Number of users (%)**	**Oral and respiratory**	**Digestive organs**	**Thyroid and lymphoid**	**Female genital**	**Male genital**	**Breast**	**Other cancers**
Agnus castus (*Vitex agnus castus)*	4 (1.8)	—	—	—	—	—	4 (100.0)	—
Black cohosh (*Cimicifuga racemosa*)	8 (3.6)	—	1 (12.5)	—	3 (38.0)	—	4 (50.0)	—
Devil's claw (*Harpagophytum procumbens*)	4 (1.8)	—	—	—	—	1 (25.0)	3 (75.0)	—
Dong quai (*Angelica sinensis*)	2 (0.9)	—	—	—	—	—	2 (100.0)	—
Echinacea (*Echinacea purpurea*)	48 (21.5)	—	3 (6.3)	2 (4.2)	9 (18.8)	5 (10.4)	29 (54.2)	—
Evening primrose (*Oenothera biennis*)	61 (27.4)	1 (1.6)	6 (9.8)	2 (3.3)	6 (9.8)	—	45 (73.8)	1 (1.6)
Garlic (*Allium sativum*)	43 (19.3)	—	6 (14.0)	3 (7.0)	5 (11.6)	8 (18.6)	21 (48.8)	—
Ginger (*Zingiber officinale*)	19 (8.5)	—	3 (15.8)	1 (5.3)	5 (26.3)	2 (10.5)	8 (42.1)	—
Gingko biloba	17 (7.6)	—	3 (17.6)	—	3 (17.6)	3 (17.6)	8 (47.1)	—
Ginseng (*Panax ginseng*)	14 (6.3)	—	1 (7.1)	—	2 (14.3)	3 (21.4)	8 (57.1)	—
Ivy (*Hedera helix*)	0 (0.0)	—	—	—	—	—	—	—
Milk thistle (*Silybum marianum*)	16 (7.2)	—	3 (18.8)	1 (6.3)	3 (18.8)	1 (6.3)	8 (50.0)	—
Mistletoe (*Viscum album*)	6 (2.7)	—	3 (50.0)	—	1 (16.7)	—	2 (33.3)	—
Red clover (*Trifolium pratense*)	5 (2.2)	—	1 (20.0)	—	1 (20.0)	1 (20.0)	2 (40.0)	—
Red vine leaf (*Vitis vinifera*)	2 (0.9)	—	—	—	—	—	2 (100.0)	—
Saw palmetto (*Serenoa serrulata*)	3 (1.3)	—	—	—	—	3 (100.0)	—	—
St John's Wort (*Hypericum perforatum*)	6 (2.7)	—	2 (33.3)	—	1 (16.7)	—	2 (33.3)	1 (16.7)
Valerian (*Valeriana officinalis*)	12 (5.4)	—	1 (8.3)	—	2 (16.7)	2 (16.7)	6 (50.0)	1 (8.3)
Wild yam (*Dioscorea villosa*)	2 (0.9)	—	—	—	—	—	2 (100.0)	—
Willow (*Salix alba*)	2 (0.9)	—	—	—	—	—	2 (100.0)	—
Any herbal medicine	274	1 (0.4)	33 (12.0)	9 (3.3)	41 (15.0)	29 (10.6)	158 (57.7)	3 (1.1)

a Percentages refer to the proportion of users of each herb from within each cancer type.

**Table 3:** Herbal medicine users’ source of information and purchase

**Source**	**Number (%)**
Taken a herbal remedy bought off the shelf in a high street chemist, supermarket, market stall, or health store	151 (67.7)
Consulted a practitioner of Western herbal medicine and taken prescribed herbs	8 (3.6)
Consulted a practitioner of Chinese/Ayurvedic herbal medicine and taken prescribed herbs	9 (4.0)
Consulted a practitioner of another herbal tradition and taken prescribed herbs	5 (2.2)
Taken a herbal remedy prescribed or recommended by GP, hospital doctor ,or other health-care professional	52 (23.3)
Taken a herbal remedy given to you by someone else	15 (6.7)
Taken a herbal remedy bought on the internet or through mail order	51 (22.9)
Used a herbal remedy from another source	6 (2.7)
Totals	223 (100.0)

Abbreviation: GP=general practitioner.

## References

[bib1] Ali N, Hussain-Gambles M (2005) Complementary and alternative medicine (CAM) use among South Asian patients with cancer in Britain. Diversity Health Social Care 2(1): 41–45

[bib2] Astin JA, Reilly C, Child WL (2006) Breast cancer patients’ perspectives on and use of complementary and alternative medicine: a study by the Susan G. Komen Breast Cancer Foundation. J Soc Integr Oncol 4(4): 157–169 17022924 10.2310/7200.2006.019

[bib3] Balneaves LG, Kristjanson LJ, Tataryn D (1999) Beyond convention: describing complementary therapy use by women living with breast cancer. Patient Educ Couns 38(2): 143–153 14528706 10.1016/s0738-3991(99)00061-0

[bib4] Cancer Backup (2010) (http://www.cancerbackup.org.uk/QAs/83586237). Accessed 15 October

[bib5] Catt SL, Fallowfield LJ, Langridge CI (2006) What non-prescription treatments do UK women with breast cancer use? Eur J Cancer Care 15(3): 279–285 10.1111/j.1365-2354.2006.00652.x 16882125

[bib6] Corner J, Harewood J, Maslin-Prothero S, Lewith G (2006) A Study of the Use of Complementary and Alternative Therapies among People Undergoing Cancer Treatment: A Quantitative and Qualitative Study. Department of Health NHS R&D Programme. Department of Health: London

[bib7] Crocetti E, Crotti N, Feltrin A, Ponton P, Geddes M, Buiatti E (1998) The use of complementary therapies by breast cancer patients attending conventional treatment. Eur J Cancer 34(3): 324–328 9640216 10.1016/s0959-8049(97)10043-0

[bib8] Department of Communities and Local Government (2007) Indices of Multiple Deprivation. CLG: London, (http://www.communities.gov.uk/publications/communities/indiciesdeprivation07). Accessed 21 July 2010.

[bib9] Downer SM, Cody MM, McCluskey P, Wilson PD, Arnott SJ, Lister TA, Slevin ML (1994) Pursuit and practice of complementary therapies by cancer patients receiving conventional treatment. BMJ 309(6947): 86–89 8038672 10.1136/bmj.309.6947.86 PMC2540590

[bib10] Ernst E (2000) Prevalence of use of complementary/alternative medicine: a systematic review. Bull World Health Organ 78(2): 252–257 10743298 PMC2560678

[bib11] Ernst E, Cassileth BR (1998) The prevalence of complementary/alternative medicine in cancer: a systematic review. Cancer 83(4): 777–782 9708945 10.1002/(sici)1097-0142(19980815)83:4<777::aid-cncr22>3.0.co;2-o

[bib12] Evans M, Shaw A, Thompson EA, Falk S, Turton P, Thompson T, Sharp D (2007) Decisions to use complementary and alternative medicine (CAM) by male cancer patients: information-seeking roles and types of evidence used. BMC Complement Altern Med 7: 25 17683580 10.1186/1472-6882-7-25 PMC2000907

[bib13] Frye R, Fitzgerald SM, Lagattuta TF, Hruska MW, Egorin MJ (2004) Effect of St John's Wort on imatinib mesylate pharmacokinetics. Clin Pharmacol Ther 76(4): 323–329 15470331 10.1016/j.clpt.2004.06.007

[bib14] Gratus C, Damery S, Wilson S, Warmington S, Routledge P, Grieve R, Steven N, Jones J, Greenfield S (2009a) The use of herbal medicines by people with cancer in the UK: a systematic review of the literature. Q J Med 102(12): 831–842 10.1093/qjmed/hcp137 19797394

[bib15] Gratus C, Wilson S, Greenfield S, Damery S, Warmington S, Grieve R, Steven N, Routledge P (2009b) The use of herbal medicines by people with cancer: a qualitative study. BMC Complement Altern Med 9: 14 19442268 10.1186/1472-6882-9-14 PMC2685766

[bib16] Harris P, Finlay IG, Cook A, Thomas KJ, Hood K (2003) Complementary and alternative medicine use by patients with cancer in Wales: a cross-sectional survey. Complement Ther Med 11(4): 249–253 15022659 10.1016/s0965-2299(03)00126-2

[bib17] Harrison A, Holt D, Pattison DJ, Elton PJ (2004) Who and how many people are taking herbal supplements? A survey of 21 923 adults. Int J Vitam Nutr Res 74(3): 183–186 15296076 10.1024/0300-9831.74.3.183

[bib18] McCune JS, Hatfield AJ, Blackburn AA, Leith PO, Livingston RB, Ellis GK (2004) Potential of chemotherapy-herb interactions in adult cancer patients. Support Care Cancer 12(6): 454–462 14991387 10.1007/s00520-004-0598-1

[bib19] Medicines and Healthcare Products Regulatory Agency (2007) Black Cohosh: UK Public Assessment Report. MHRA: London

[bib20] Miller M, Boyer MJ, Butow PN, Gattellari M, Dunn SM, Childs A (1998) The use of unproven methods of treatment by cancer patients: frequency, expectations and cost. Support Care Cancer 6(4): 337–347 9695201 10.1007/s005200050175

[bib21] Molassiotis A, Margulies A, Fernandez-Ortega P, Pud D, Panteli V, Bruyns I, Scott JA, Gudmundsdottir G, Browall M, Madsen E, Ozden G, Magri M, Selvekerova S, Platin N, Kearney N, Patiraki E (2005) Complementary and alternative medicine use in patients with haematological malignancies in Europe. Complement Ther Clin Pract 11(2): 105–110 15955292 10.1016/j.ctcp.2004.12.005

[bib22] Morris KT, Johnson N, Homer L, Walts D (2000) A comparison of complementary therapy use between breast cancer patients and patients with other primary tumor sites. Am J Surg 179(5): 407–411 10930491 10.1016/s0002-9610(00)00358-5

[bib23] National Cancer Intelligence Network (2009) Cancer Incidence and Survival by Major Ethnic Group, England, 2002–2006. National Cancer Intelligence Network in collaboration with the Cancer Research UK Cancer Survival Group, London School of Hygiene and Tropical Medicine: London

[bib24] Olaku O, White JD (2010) Herbal therapy use by cancer patients: a literature review on case reports. Eur J Cancer (in press), doi:10.1016/j.ejca.2010.11.018 10.1016/j.ejca.2010.11.018 PMC3057114 21185719

[bib25] Office for National Statistics (2006) Cancer Registrations in England. ONS: London (http://www.statistics.gov.uk/downloads/theme_health/2006cancerfirstrelease.xls). Accessed 4 September 2010

[bib26] Rees RW, Feigel I, Vickers A, Zollaman C, McGurk R, Smith C (2000) Prevalence of complementary therapy use by women with breast cancer: a population based survey. Eur J Cancer 36(11): 1359–1364 10899648 10.1016/s0959-8049(00)00099-x

[bib27] Saxe GA, Madlensky L, Kealey S, Wu DP, Freeman KL, Pierce JP (2008) Disclosure to physicians of CAM use by breast cancer patients: findings from the Women's Healthy Eating and Living Study. Integr Cancer Ther 7(3): 122–129 18956493 10.1177/1534735408323081 PMC2763208

[bib28] Verhoef MJ, Hilsden RJ, O’Beirne M (1999) Complementary therapies and cancer care: an overview. Patient Education Counseling 38(2): 93–100 14528701 10.1016/s0738-3991(99)00056-7

[bib29] Vickers KA, Jolly KB, Greenfield SM (2006) Herbal medicine: women's views, knowledge and interaction with doctors: a qualitative study. BMC Complement Altern Med 6: 40 17156416 10.1186/1472-6882-6-40 PMC1702550

[bib30] Werneke U, Earl J, Setdel C, Horn O, Crichton P, Fannon D (2004a) Potential health risks of complementary alternative medicines in cancer patients. Br J Cancer 90(2): 408–413 14735185 10.1038/sj.bjc.6601560 PMC2410154

[bib31] Werneke U, Ladenheim D, McCarthy T (2004b) Complementary alternative medicine for cancer: a review of effectiveness and safety. Cancer Ther 2: 475–500

[bib32] World Health Organization (1994) International Statistical Classification of Diseases and Related Health Problems, 10th Revision (ICD-10). (http://apps.who.int/classifications/apps/icd/icd10online/). Accessed 21 July 2010

